# The role of arts therapies in mitigating Sleep Initiation and Maintenance Disorders: a systematic review

**DOI:** 10.3389/fpsyt.2024.1386529

**Published:** 2024-05-16

**Authors:** Xuexing Luo, Aijia Zhang, Hong Li, Yu Li, Fangtian Ying, Xiaoli Wang, Qianxu Yang, Zheyu Zhang, Guanghui Huang

**Affiliations:** ^1^ Faculty of Humanities and Arts, Macau University of Science and Technology, Macao, Macao SAR, China; ^2^ State Key Laboratory of Quality Research in Chinese Medicines, Macau University of Science and Technology, Macao, Macao SAR, China; ^3^ Faculty of Chinese Medicine, Macau University of Science and Technology, Macao, Macao SAR, China; ^4^ College of Computer Science and Technology Zhejiang University, Hangzhou, Zhejiang, China; ^5^ Operation Management Centre, Guangzhou Wanqu Cooperative Institute of Design, Guangzhou, Guangdong, China; ^6^ Qinghai Province Cardiovascular and Cerebrovascdular Disease Specialist Hospital, Xining, Qinghai, China; ^7^ Centre for Epidemiology and Evidence-Based Practice, Department of Social and Preventive Medicine, Faculty of Medicine, University of Malaya, Kuala Lumpur, Malaysia

**Keywords:** Arts Therapies, sleep disorders, psychotherapy, complementary interventions, mental health

## Abstract

**Introduction:**

Arts therapies offer effective non-pharmacological intervention for Sleep Initiation and Maintenance Disorders (SIMDs), encompassing both passive and active modalities. This review assesses their effectiveness and ethical considerations, focusing on music therapy, meditation, and Tai Chi.

**Methods:**

Following PRISMA guidelines, a detailed search across PubMed, the Cochrane Library, Web of Science, and CNKI identified 17 relevant RCTs. Utilizing the Joanna Briggs Institute (JBI) quality criteria and the PICO(S) framework for data extraction ensured methodological integrity.

**Results:**

Analysis shows arts therapies significantly improve sleep quality. Music therapy and meditation yield immediate benefits, while Tai Chi and Qigong require longer commitment for significant outcomes.

**Discussion:**

The link between SIMDs and mental health issues like anxiety, stress, and depression suggests arts therapies not only enhance sleep quality but also address underlying mental health conditions. The evidence supports a wider adoption of arts therapies in treating SIMDs due to their dual benefits.

**Systematic review registration:**

PROSPERO, ID: CRD42024506393.

## 1 Introduction

In the rapid pace of modern life, Sleep initiation and maintenance disorders (SIMDs) have emerged as a global health challenge, exacerbated by the aftereffects experienced by many in the wake of the pandemic, severely impacting physical health and daily routines (1). A study by the World Health Organization (WHO) estimates that approximately 1.7 billion people globally suffer from sleep disturbances, accounting for 27% of the world’s population (2). The Diagnostic and Statistical Manual of Mental Disorders (DSM-5) characterizes insomnia as a prevalent clinical condition, with approximately one-third of adults reporting symptoms of insomnia, and 6 to 10% meeting the diagnostic criteria for an insomnia disorder (3). SIMDs encompass a variety of abnormalities during sleep, including excessive daytime sleepiness, insomnia, abnormal movements or behaviors during sleep, and difficulty initiating sleep when desired (4, 5), often accompanied by other physical conditions or mental health issues, including insomnia, obstructive sleep apnea, somnambulism, narcolepsy, and restless legs syndrome among different types (6, 7). More than 50% of adults globally have experienced a sleep disorder at least once in their lives (8). The distribution of sleep problems across certain age groups and populations is even broader, becoming a significant and growing public health issue (9). Sleep, as a fundamental human need, plays a crucial role in maintaining overall health, cognitive function, and quality of life (10). Research indicates that most SIMDs are preventable or treatable (11), yet the majority of sufferers do not seek professional help. The consequences of SIMDs are wide-ranging, adversely affecting individual health and socioeconomic well-being. Conditions such as insomnia, obstructive sleep apnea (OSA), and restless legs syndrome (RLS) continue to have high prevalence rates and exert profound effects on physical and mental health (12–14). Studies have shown a close correlation between sleep and an increased risk of obesity, diabetes, cardiovascular diseases, and mental health disorders, including depression and anxiety (15–17). Sleep issues not only have a strong bidirectional relationship with mood disorders such as depression and anxiety but are also associated with cognitive decline, physical health problems, and decreased work efficiency (18–21). Particularly among the elderly, over 50% suffer from sleep issues, impacting their physical and mental well-being (22). Furthermore, approximately 80% of individuals with clinical depression experience sleep disturbances (23). Research also reveals that sleep problems are prevalent in high-stress groups, such as college students (24). Traditional sleep interventions have relied on pharmacological treatments, which may come with side effects that limit their long-term application (25), such as residual drowsiness, tolerance, dependence, altered sleep architecture, and rebound insomnia (26–29). Common medications used in traditional treatments include sleep aids like zopiclone, zolpidem, and eszopiclone, as well as medications used to alleviate anxiety or depression associated with sleep issues, such as alprazolam, quetiapine, and fluoxetine (30, 31). These medications often result in side effects during the medication-induced sleep process, such as nasal congestion, convulsions, nightmares or hallucinations, and breathing difficulties, and are accompanied by side effects such as dizziness, nausea, dry mouth, muscle weakness, and headache upon waking. Moreover, these drugs carry the risk of dependency and resistance, often leading to withdrawal reactions upon cessation, and place significant financial stress on many patients over the long term (32, 33). This has paved the way for non-pharmacological interventions (34), given the burdens of pharmacotherapy, exploring benign non-pharmacological interventions for sleep issues becomes the focus of this article.

Among the numerous methods for sleep healing, various forms of arts therapies as non-pharmacological treatments have shown unique advantages and potential. To refine and expand our definition of arts within arts therapies, it’s crucial to adopt a nuanced and comprehensive perspective that acknowledges the wide array of non-pharmacological therapeutic arts as defined by the five senses: visual, auditory, olfactory, gustatory, and tactile. For example, visual art and color therapy utilize form, color, and imagery to engage the visual sense, potentially evoking feelings of calmness and relaxation that can improve sleep quality (35). Similarly, music therapy leverages auditory stimuli to influence emotional states and stress levels, indirectly promoting healthier sleep patterns (36). Aromatherapy, targeting the olfactory sense, uses essential oils and fragrant extracts to harmonize the mind and body, with certain scents known for their sedative properties that aid sleep. While gustatory arts, such as culinary arts therapy, may indirectly influence sleep through mood and wellbeing improvements, tactile forms of therapy like body movement and dance therapy emphasize physical expression and the tactile experience to relieve stress and enhance sleep quality (37, 38). This inclusive approach not only broadens the review’s scope but also underscores the multifaceted nature of arts therapies in mitigating sleep initiation and maintenance disorders by alleviating mental stress and emotional tension. Moreover, arts therapies promote and encourage individuals to actively manage their own health, placing a greater emphasis on intrinsic motivation and personal involvement. By engaging with art, individuals improve their mood and mental health, releasing inner stress and uneasy emotions, which in turn reduces levels of anxiety and depression (39). This engagement in creative activities provides an outlet for emotional release and self-exploration, not only offering immediate relief from sleep issues but also increasing individual life satisfaction and happiness over the long term. As a non-pharmacological treatment, arts therapies not only highlights its uniqueness in addressing sleep problems but also robustly supports the improvement of sleep quality, promising to become an important tool for enhancing individual and community health (40).

Therefore, this review explores a gentler approach to improving sleep health through non-pharmacological interventions. Focusing on various art forms, this review examines diversified alternative therapies including mindfulness-based stress reduction (MBSR) (41), yoga (42), music therapy (43), virtual reality (VR) (44), and meditation (45) that offer sensory engagement. Studies indicate that these therapies assist in regulating both physiological and psychological states, thus improving sleep quality. These arts therapies not only eliminate the need for injections or oral medications but also provide multidimensional benefits unreachable by traditional treatment methods (46). For instance, group yoga not only promotes relaxation and improves sleep quality but also enhances social interaction, boosts self-esteem, and positively affects posture (47). Beyond sleep, these interventions also include stress reduction, enhanced emotional regulation, and improved cognitive function (48, 49). These intervention methods offer assistance not just to the elderly but have also shown potential in ICU patients, college students, and other populations affected by high stress levels.

This review aims to comprehensively examine and compare evidence supporting various non-pharmacological interventions for SIMDs, delving into how these measures can improve sleep issues across different populations and assessing their benefits and potential limitations in clinical practice. Specifically, this review will focus on the following research questions:

1) Which arts therapies have been effective in addressing SIMDs?
2) How do different art forms and practices leverage their unique advantages in the intervention of SIMDs?
3) What are the mechanisms of effect when these arts therapies are used as alternative treatments, and what hypotheses can be explored?

This review intends to provide healthcare professionals with practical guidelines to make more informed decisions in treating SIMDs and offer value to patients seeking alternative and complementary treatments. By considering the multidimensional benefits of arts therapies, this article aims to present a new perspective and direction for the management of SIMDs within the healthcare system.

## 2 Methods

This systematic review adheres to the PRISMA statement for systematic reviews and meta-analyses (PRISMA) (50, 51) and is registered with PROSPERO (52) under the registration number CRD42024506393. We developed the research question using the PICOS acronym, as follows:

PICOS

• P: Any patient associated with SIMDS.
• I: Interventional arts therapies.
• C: Conventional therapy.
• O: Improved clinical and/or mental health outcomes.
• S: randomized controlled trials, quasi experimental studies (non-randomized controlled trials), and single-arm, pre-test/post-test studies.

### 2.1 Study inclusion and exclusion criteria

In the systematic review, we rigorously defined the inclusion and exclusion criteria to ensure a thorough examination of the impact of arts therapies on Sleep Initiation and Maintenance Disorders (SIMDs). Studies eligible for inclusion are those that adhere to the Joanna Briggs Institute (JBI) research design guidelines, involve participants diagnosed with SIMDs, and implement arts therapies, including but not limited to music, visual arts, dance/movement, drama therapy, and bibliotherapy as a primary intervention. The interventions could be carried out across various settings and delivered by arts therapy professionals. These studies should compare the outcomes against any type of control group and report on sleep quality indicators such as sleep latency and efficiency. Our focus is on gathering empirical evidence from randomized controlled trials, quasi-experimental studies, and analytical cross-sectional studies that meet ethical and methodological standards.

Conversely, the review excludes non-peer-reviewed documents, studies not employing art-based interventions, and those concerning individuals without SIMDs diagnoses. Additionally, research designs such as non-experimental studies, narrative reviews, animal studies, and publications not in English are omitted to maintain the review’s integrity and manageability. This selectivity ensures the inclusion of studies with robust methodologies and relevant outcomes, thereby providing reliable evidence on the effectiveness of arts therapies in treating sleep disorders. The exclusion of non-English articles and grey literature is acknowledged as a limitation but is necessary for ensuring thorough analysis and interpretation within the language proficiency of the review team.

### 2.2 Electronic databases

This systematic search was conducted across five electronic databases: PubMed, Cochrane Library, Web of Science, Embase, and the Chinese database CNKI, for publications spanning from 2004 to 2024.

### 2.3 Search strategy

A meticulously structured search strategy was implemented by a team of three independent researchers. This strategy aimed to capture a wide array of studies that investigate the intersection of arts therapies and sleep disorders. **Table 1** of the original article delineates the search strategy, highlighting the employment of specific keywords and Medical Subject Headings (MeSH) to guide the literature search. The primary keywords included ‘Arts Therapies’ and ‘Sleep Disorders,’ which were chosen for their broad applicability to studies exploring the therapeutic use of art in the context of sleep-related issues.

**Table 1:** Search strategies for English databases or Chinese databases.

Number	Search Terms
#1	Music Therapy [MeSH]
#2	Color Therapy [MeSH]
#3	Art Therapy [MeSH]
#4	Meditation [MeSH]
#5	Mindfulness [MeSH]
#6	Tai ji [MeSH]
#7	Qigong [MeSH]
#8	Yoga [MeSH]
#9	Exercise Movement Techniques [MeSH]
#10	Dance Therapy [MeSH]
#11	Virtual Reality [MeSH]
#12	Play Therapy [MeSH]
#13	Psychodrama [MeSH]
#14	Drawing [MeSH]
#15	#1 OR #2 OR #3 OR #4 OR #5 OR #6 OR #7 OR #8 OR #9 OR #10 OR #11 OR #12 OR #13 OR #14
#16	Sleep Initiation and Maintenance Disorders [MeSH]
#17	Insomnia [MeSH]
#18	Sleep [MeSH]
#19	#16 OR #17 OR #18
#20	#15 AND #19
#21	Yinyue liaofa (Music Therapy)
#22	Secai liaofa (Color Therapy)
#23	Yishu liaofa (Art Therapy)
#24	Minxiang liaofa (Meditation)
#25	Zhengnian liaofa (Mindfulness)
#26	Tai ji (Tai ji)
#26	Qigong (Qigong)
#27	Yujia (Yoga)
#28	Yundong (Exercise Movement Techniques)
#29	Wudao liaofa (Dance Therapy)
#30	Xunixiangshi (Virtual Reality)
#31	Youxi liaofa (Play Therapy)
#32	Xiju liaofa (Psychodrama)
#33	Huihua (Drawing)

MeSH, Medical Subject Headings.

To ensure a comprehensive gathering of relevant literature, various forms of art therapy were considered, encompassing visual arts and color-related therapies, auditory music-related therapies, and tactile movement-related therapies. This approach acknowledges the multifaceted nature of arts therapies and their potential impact on sleep disorders across different sensory modalities. Furthermore, to mitigate the risk of overlooking pertinent studies, a manual search was conducted through the reference lists of key reviews and articles identified during the initial search phase. This dual-faceted search strategy, combining both electronic database searches with manual reference checking, was aimed at creating a thorough and all-encompassing review of the literature available on the subject matter, enhancing the reliability and depth of the systematic review.

### 2.4 Study selection

Three independent reviewers, LXX, HL, and AJZ, engaged in the screening, eligibility, and selection review processes. LXX was tasked with downloading and reviewing the filtered articles, excluding those deemed irrelevant. Subsequently, pertinent literature was forwarded to HL for an eligibility review. Upon determining the eligibility of 17 documents, AJZ conducted a meticulous evaluation and scoring based on JBI’s critical appraisal tools (53, 54). These instruments are designed to assess the methodological quality of the studies and ascertain the extent to which the studies address the potential for bias in their design, execution, and analysis. Moreover, literature meeting the eligibility criteria was subjected to a dual examination by LXX and HL. Every one of the 17 included studies, particularly those identified as randomized controlled trials, underwent a thorough data extraction process using the PICO(S) framework (55). This approach allowed for the systematic organization and assessment of pertinent information extracted from the documents, enhancing the clarity and conciseness of the analysis.

### 2.5 Data extraction

In the systematic review process, the task of data extraction was meticulously planned and executed by three independent researchers, ensuring a thorough and unbiased collection of data from the selected studies. This critical phase was structured around the creation of four distinct tables, each designed with a specific function to aid in the systematic organization and analysis of the collected data.

The first table was developed to provide a clear visualization of the core content extracted from each piece of literature, organized into categories such as Study Aims, Participants, Methods, Results, and Discussion. This table aimed to facilitate an immediate understanding of each study’s key components, allowing for a quick yet comprehensive overview of the collected data. The second table was tailored to display subgroups of different arts therapies, enabling the researchers to categorize and compare the results and author names within specific therapeutic groups, thereby highlighting the diversity and specific outcomes associated with each art therapy type.

To ensure the quality and reliability of the included studies, a third table was utilized for conducting a quality review of the literature. This table employed checklists derived from the Joanna Briggs Institute (JBI) guidelines, which are renowned for their robustness in assessing the methodological quality of research. The fourth table was dedicated to extracting detailed data about patient samples, intervention and control group methodologies, and outcomes, specifically from randomized controlled trials (RCTs). For this purpose, the researchers adopted the PICOS (Population, Intervention, Comparator, Outcomes, and Study Design) model, which facilitated a structured and comprehensive analysis of the RCTs. This strategic approach to data extraction not only enhanced the clarity and organization of the data but also laid a solid foundation for the systematic review’s subsequent analysis and discussions.

### 2.6 Quality appraisal of the included studies

The initial comprehensive search yielded 17,262 publications, from which irrelevant studies were excluded through a meticulous screening of titles and abstracts, followed by a detailed full-text review as required. This rigorous selection process leveraged the PICO(S) framework (Population, Intervention, Comparison, Outcome, and Study design) to systematically define inclusion and exclusion criteria, ensuring focus and relevance in the selection of studies, to uphold the methodological integrity of the review, the Joanna Briggs Institute (JBI) critical appraisal tools were employed, facilitating the evaluation of the studies’ quality. This approach underlined our commitment to incorporating only the most methodologically sound literature into our analysis (53–56).

The quality appraisal of randomized controlled trials (RCTs) within our dataset was conducted using a scoring system informed by JBI guidelines. This involved assigning a score of one point for each criterion adequately met ‘yes’, zero points for unmet criteria ‘no’ and ‘unclear’. This scoring facilitated a horizontal comparison of study quality, ranking them based on their aggregate scores. A score of ≤6 was considered as low quality, from 7 to 9 as moderate and ≥ 10 as high quality. No studies were excluded based on methodological quality. Vertically, this method allowed for the assessment of commonalities across the reviewed literature, evaluating the proportion of studies that successfully met each quality criterion.

Furthermore, the application of the PICO(S) framework extended beyond initial study selection, informing the structured tabulation of the research data. This included organizing studies and ranking them by the number of participants, recognizing that larger sample sizes typically contribute to a stronger evidence base.

## 3 Results

### 3.1 Study characteristics

**Figure 1:**
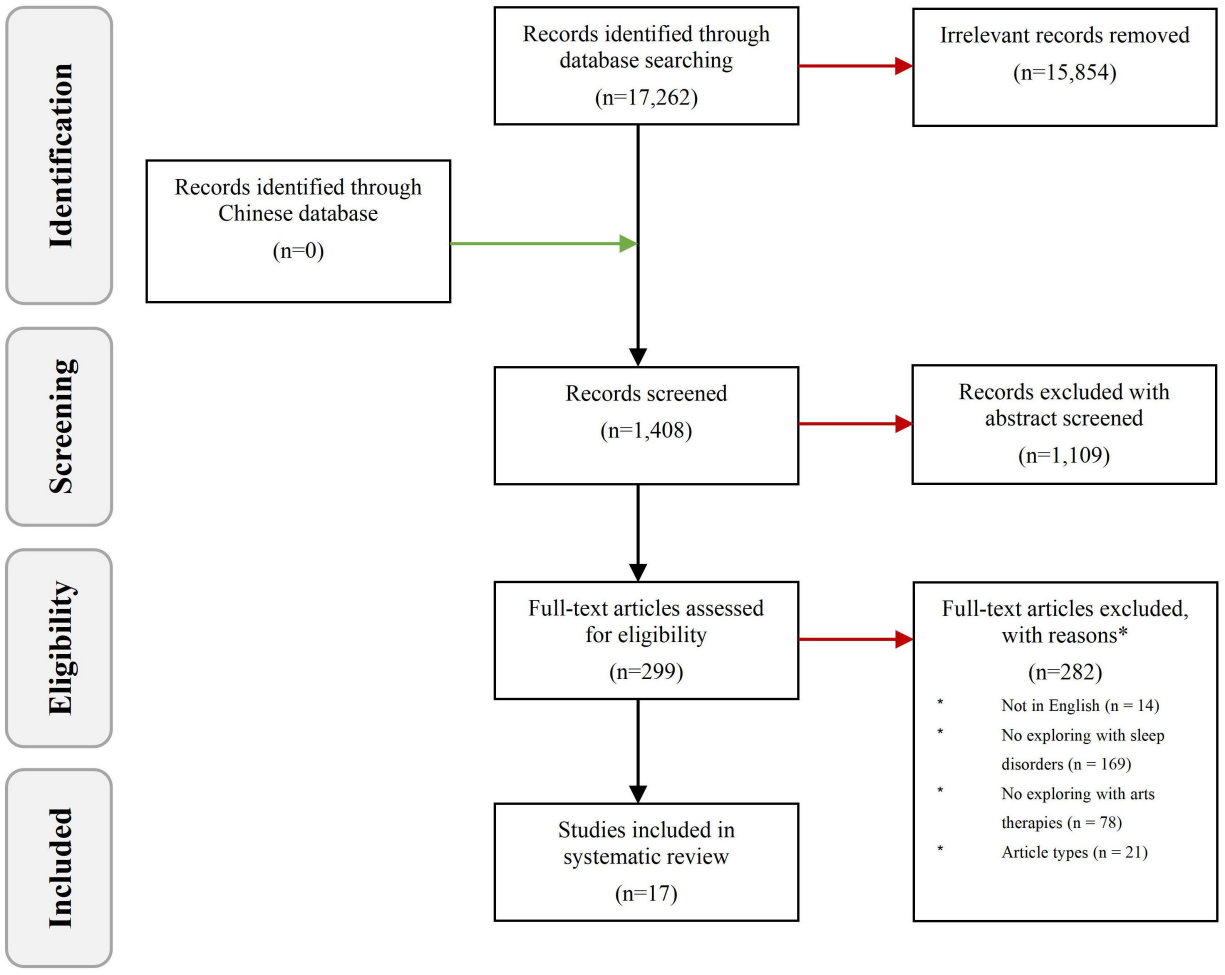
Flow diagram for the included and excluded articles.

**Figure 2:**
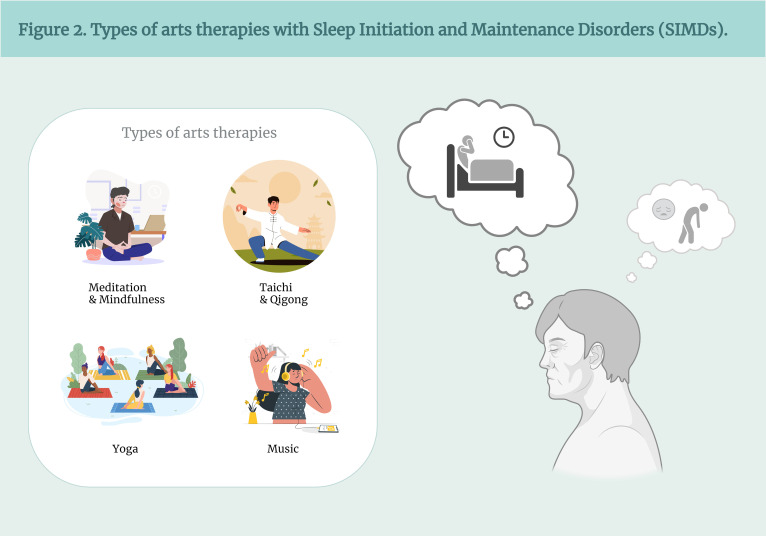
Types of arts therapies with Sleep Initiation and Maintenance Disorders (SIMDs).

This manuscript incorporates 17 studies focusing on experimental design, wherein 10 studies juxtapose art therapy with a standard routine, and three compare art therapy to sleep hygiene education, see in **Figure 1**. The control groups in other investigations employed a variety of methods including daily routine sleep intervention, self-monitoring, low-impact exercise, and health education, see in **Figure 2**, primarily encompassing Virtual Reality (VR) (57), meditation (58–60), qigong (61), tai chi (62, 63), Biodanza (64), music therapy (65–70), yoga (71, 72), and Mindfulness-Based Stress Reduction (MBSR)/Mindfulness-Based Cognitive Therapy (MBCT) (73).

**Tables 2**, **3** synthesize the principal characteristics of the qualifying studies. The 17 included studies comprise 1,300 participants (treatment conditions= 694, control conditions= 606) hailing from South Korea (57), the USA (58–60, 62, 63, 71, 72), China (61, 65–67, 70), Spain (64), Hungary (68), Singapore (69), and Iran (73), with the largest sample consisting of 237 individuals (58) and the smallest of 18 (71). The age range of participants was broad, from teenagers aged 19 to senior citizens aged 92, encompassing healthy individuals (61–63, 66, 69, 72), cardiac patients (57), those with chronic insomnia and moderate SIMDs (58–60, 65–68, 70, 71), stressed university students (64), and individuals with depression (73).

**Table 2:** Record of citations and full text reviewed.

Name	Types	Region	study aims	Participants	Methods	Results	Discussion
Soon Young, et al. (57)	RCT	South Korea	investigate the effect of virtual reality meditation on sleep quality of intensive care unit patients.	48 cardiac intensive care unit patients in a university hospital in Korea randomly allocated to the experimental (24) and the control group (24).	meditation was provided for 30 minutes using a head-mounted display for virtual reality, on the evening of the admission day.	the awake time was shorter, deep sleep time was longer and sleep efficiency was significantly higher in the experimental group than in the control group.	Although VR meditation did not improve total sleep time (TST) and light sleep time, it did enhance the overall quality of sleep.
Jennifer, et al. (58)	RCT	USA	1) determine the effects of a meditation app on depression and anxiety in adults with sleep disturbance, and 2) explore the potential mediating effects of fatigue, daytime sleepiness, and pre-sleep arousal on the relationship between use of the meditation app and changes in depression and anxiety.	239 adults with elevated insomnia symptoms (i.e., scores ≥ 10 on the Insomnia Severity Index) and limited or no previous experience with meditation.	Depression, anxiety, fatigue, daytime sleepiness, and pre-sleep arousal were assessed at baseline, four weeks, and eight weeks. Repeated-measures ANCOVAs assessed intervention effects on depression and anxiety.	Participants in the meditation group had more improvement in depression and anxiety symptoms during the intervention period than did those in the control group	The study emphasizes the importance of pre-sleep arousal as a potential target for intervention in treating mental health issues.
Jason, et al. (59)	RCT	USA	evaluate the efficacy of mindfulness meditation for the treatment of chronic insomnia.	54 adults with chronic insomnia, Participants were randomized to either mindfulness-based stress reduction (MBSR).	8-week group intervention that includes weekly 2.5-hour sessions, plus a 6-hour meditation retreat held between the 5th and 7th week.	Participants who received mindfulness meditation interventions (either MBSR or MBTI) showed significantly greater reductions in total wake time (TWT) in bed, pre-sleep arousal (PSAS), and clinically significant changes in treatment response and remission compared to the self-monitoring (SM) control group.	The effect sizes observed in this study are generally like those reported in behavioral clinical trials on chronic insomnia, providing preliminary support that meditation-based treatments are viable non-pharmacological treatments for adults with insomnia.
David, et al. (60)	RCT	USA	determine the efficacy of a mind-body medicine intervention, called mindfulness meditation, to promote sleep quality in older adults with moderate sleep disturbances.	Randomized clinical trial with 2 parallel groups conducted among an older adult sample (mean [SD] age, 66.3 [7.4] years) with moderate sleep disturbances (Pittsburgh Sleep Quality Index [PSQI] >5).	mindful awareness practices (MAPs) intervention (n = 24) or a sleep hygiene education (SHE) intervention (n = 25) was randomized to participants, who received a 6-week intervention (2 hours per week) with assigned homework.	The MAPs group showed significant improvement relative to the SHE group on secondary health outcomes of insomnia symptoms, depression symptoms, fatigue interference, and fatigue severity (P <.05 for all).	The study suggests that mindfulness meditation could be a promising short-term treatment for sleep issues in this population, with potential mechanisms involving cognitive and neurocognitive processes.
Shu-chuan, et al. (61)	RCT	Taiwan	examine the effect of a 12-week 30-minute-a-day Ping Shuai Qigong exercise program on climacteric symptoms and sleep quality in perimenopausal women.	Thirty-five (35) women from one community were assigned to a Ping Shuai Qigong intervention group, while 35 women from the other community were assigned to the control group	12-week, 30-minute-a-day Ping Shuai Qigong program, Descriptive analysis and repeated-measures analysis of variance were used.	found to have significant improvements in sleep quality in those times.	the program appears to have a greater effect on sleep latency, habitual sleep efficiency, and sleep disturbance than on subjective sleep quality, sleep duration, and daytime dysfunction.
Fuzhong, et al. (62)	RCT	USA	this program appears to have a greater effect on sleep latency, habitual sleep efficiency, and sleep disturbance than on subjective sleep quality, sleep duration, and daytime dysfunction.	One hundred eighteen women and men aged 60 to 92.	Participants were randomized into tai chi or low-impact exercise and participated in a 60-minute session, three times per week, for 24 consecutive weeks.	older adults with moderate sleep complaints who participated in a tai chi program reported significant improvements in self-rated sleep quality and daytime sleepiness compared to those in a low-impact exercise group.	tai chi participants experienced better physical performance and quality of life compared to the low-impact exercise group.
Michael, et al. (63)	RCT	USA	determine the efficacy of a novel behavioral intervention, Tai Chi Chih, to promote sleep quality in older adults with moderate sleep complaints.	Volunteer sample of 112 healthy older adults, aged 59 to 86 years.	Random allocation to Tai Chi Chih or health education for 25 weeks.	Tai Chi Chih (TCC) can be considered a useful nonpharmacologic approach to improve sleep quality in older adults with moderate sleep complaints.	TCC training is related to improvements in self-rated sleep quality among older adults with moderate sleep complaints.
Marı´a, et al. (64)	RCT	Spain	determine the effectiveness of Biodanza in reducing symptoms of perceived stress and depression and in promoting sleep quality in young adults, comparing the changes with those observed in a control group.	121 university students with perceived stress were randomly placed into either a Biodanza group or a wait-list control group.	Study participants attended Biodanza sessions for 90 min a week, over a period of 4 weeks.	no significant differences in sleep quality were observed between the groups (p = 0.666).	the Biodanza group reported a significant reduction in stress and depression levels compared to the control group, which showed a slight worsening of stress levels.
Chiung-Yu, et al. (65)	RCT	Taiwan	compare the effects of music and music video interventions on objective and subjective sleep quality in adults with sleep disturbances.	71 adults who were recruited from the outpatient department of a hospital with 1100 beds and randomly assigned to the control, music, and music video groups.	During the 4 test days (Days 2–5), for 30 min before nocturnal sleep, the music group listened to Buddhist music and the music video group watched Buddhist music videos.	the music group reported a significantly longer subjective total sleep time (TST) compared to the music video group (p = 0.04).	The study’s findings suggest that while music and music video interventions did not improve objective sleep parameters, listening to music before bedtime did improve subjective TST in adults with sleep disturbances.
Qun, et al. (66)	RCT	China	examine the effects of music intervention on sleep quality in community-dwelling elderly people.	64 elderly people with a mean age of 69.38 – 5.46 years were randomly assigned to the control group (n = 32) or the intervention group (n = 32).	Each participant in the intervention group received an MP3 player with a music database. The participants selected the preferred music and listened for 30–45 minutes per night for 3 months.	The intervention group showed continuous improvements in sleep quality, with significant reductions in global PSQI scores from baseline to 3 months.	music intervention is a safe and effective nonpharmacological approach for improving sleep quality in community-dwelling elderly people, particularly in reducing sleep latency, improving sleep efficiency, and reducing daytime dysfunction.
En-Ting, et al. (67)	RCT	Taiwan	evaluate the effect of soothing music on objective and subjective sleep quality in adults with chronic insomnia.	Fifty participants were enrolled in a randomized controlled trial conducted in the sleep laboratory of a hospital, with 25 participants allocated to the music group and 25 to the control group.	For four days, the experimental group was exposed to soothing music selected by the participants or researchers for 45 min at nocturnal sleep time, whereas the control group was not exposed to music.	After controlling for baseline data, the music group had significantly better scores for rested rating, shortened stage 2 sleep, and prolonged REM sleep compared to the control group.	Listening to soothing music at nocturnal sleep time improved the rested rating scores, shortened stage 2 sleep, and prolonged REM sleep, but had little effect on sleep quality as measured by PSG and self-reported questionnaires.
La´szlo´, et al. (68)	RCT	Hungary	investigate the effects of music on sleep quality in young participants with poor sleep.	94 students (aged between 19 and 28 years) with sleep complaints.	Participants listened for 45 minutes either to relaxing classical music (Group 1) or an audiobook (Group 2) at bedtime for 3 weeks.	music significantly improved sleep quality (P < 0.0001), Sleep quality did not improve significantly in the audiobook and control groups.	relaxing classical music is an effective intervention in reducing sleeping problems. The results suggest that music can improve sleep quality and depressive symptoms in young adults with poor sleep.
Angela, et al. (69)	RCT	Singapore	examine the effects of music listening on sleep quality among older community dwelling adults in Singapore.	a cohort of older adults (N = 60) age 55 years or above.	Participants were asked to listen to soft, instrumental slow sedative music without lyrics, of approximately 60—80 beats per minute, and 40 min in duration, for 6 weeks.	Significant reductions in PSQI scores were found in the intervention group from baseline to week 6, with a mean decrease from 10.2 to 5.9 (p < 0.001), while the control group showed no changes.	healthcare professionals could engage elderly clients in music listening therapy to improve sleep quality, individualizing, and enhancing the quality of care provided.
Hui-Ling, et al. (70)	RCT	Taiwan	investigation of the effects of soft music on sleep quality in older community-dwelling men and women in Taiwan.	Sixty people aged 60–83 years with difficulty in sleeping were recruited through community leaders and screened using the Pittsburgh Sleep Quality Index (PSQI) and Epworth Sleepiness Scale.	Participants listened to their choice among six 45-minute sedative music tapes at bedtime for 3 weeks.	music significantly improved sleep quality in the experimental group, with better perceived sleep quality, longer sleep duration, greater sleep efficiency, shorter sleep latency, less sleep disturbance, and less daytime dysfunction (P values ranging from 0.04 to 0.001).	music can induce relaxation and distraction responses, leading to decreased activity in the neuroendocrine and sympathetic nervous systems, which in turn can reduce anxiety, heart rate, respiratory rate, and blood pressure, and improve sleep.
Erica, et al. (71)	RCT	USA	examine trial feasibility plus physiological and psychological effects of a guided meditation practice, Yoga Nidra, in adults with self-reported insomnia.	22 adults with self-reported insomnia were recruited.	half of participants were randomized to practice Yoga Nidra for the first 30-min.	Yoga Nidra produced sleep in 89% of participants during and after the practice, and the participants reported feeling more relaxed after the intervention.	the lack of significant changes in EEG alpha power and HRV parameters indicates that further research is needed to confirm the specific mechanisms through which Yoga Nidra may facilitate relaxation and sleep.
Sat Bir, et al. (72)	RCT	USA	Prior studies have suggested a benefit of yoga for alleviating sleep disturbance; however, many studies have had methodological limitations. This trial study aimed to extend that literature by including an active sleep hygiene comparison.	a total of 44 participants were randomly assigned to either the yoga group or the sleep hygiene (SH) treatment group, with 22 participants in each group.	The yoga intervention included Kundalini yoga practices with a focus on meditation, relaxation, and breathing techniques.	The yoga group showed significant improvements in SOL, TST, and SE, with medium-to-large effect sizes.	The yoga intervention demonstrated greater overall improvements compared to the active SH intervention, suggesting that yoga may be a valuable addition to cognitive behavioral therapy for insomnia (CBT-I) or an initial treatment option in a stepped care approach.
Nima, et al. (73)	RCT	Iran	determine the effect of mindfulness-based stress reduction program on emotion regulation and sleep problems in depressed elderly.	60 elderly individuals with depression using purposive sampling.	The MBSR sessions were held for the experimental group in 8 sessions of 90 min each, once a week.	significant reduction in depression symptoms (p < 0.001) and improvement in emotion regulation and sleep quality (p < 0.001) among the elderly participants with depression in the intervention group.	significant reduction in depression symptoms (p < 0.001) and improvement in emotion regulation and sleep quality (p < 0.001) among the elderly participants with depression in the intervention group.

SOL, sleep onset latency; TST, total sleep time; SE, sleep efficiency; EEG, Electroencephalogram; PSQI, Pittsburgh sleep quality index; PSG, polysomnography; REM, rapid eye movement; SH, sleep hygiene; MBSR, Mindfulness-Based Stress Reduction.

**Table 3:** Principal characteristics of all included RCTs in this review.

Reference	ID	Design	Sample Size T/C (whole)	Sample Ranking	Outcomes Measure	Treatment group		Control Group
						Intervention	Procedure Times	
Soon Young, et al.	1 (57)	RCT	24/24 (48)	15	SSA/ATFC2/PSQI	VR	30 min	DRSI
Jennifer, et al.	2 (58)	RCT	124/113 (237)	1	HADS	Meditation	8 weeks	NR
Jason, et al.	3 (59)	RCT	19/19/16* (54)	12	TWT/PSAS/ISI	MBSR/MBTI	8 weeks	SM
David, et al.	4 (60)	RCT	24/25 (49)	14	PSQI	MAPs	6 weeks	SHE
Shu-chuan, et al.	5 (61)	RCT	35/35 (70)	7	PSQI	Qigong	12 weeks	NR
Fuzhong, et al.	6 (62)	RCT	62/56 (118)	2	PSQI/ESS	Tai Chi	2 months	L-IE
Michael, et al.	7 (63)	RCT	59/53 (112)	3	PSQI	Tai Chi Chih	25 weeks	HE
Marı´a, et al.	8 (64)	RCT	42/53 (95)	4	PSQI/PSS/CES-D	Biodanza	4 weeks	NR
Chiung-Yu, et al.	9 (65)	RCT	23/24/24* (71)	6	STAI/PSQI/EEG	M/MV	6 days	NR
Qun, et al.	10 (66)	RCT	32/32 (64)	8	PSQI	Music	3 months	SHE
En-Ting, et al.	11 (67)	RCT	25/25 (50)	13	PSQI/PSG	Music	3 days	NR
La´szlo´, et al.	12 (68)	RCT	35/30/29* (94)	5	PSQI/ESS/BDI	Music/Audiobook	3 weeks	NR
Angela, et al.	13 (69)	RCT	28/32 (60)	9	PSQI	Sedative music	6 weeks	NR
Hui-Ling, et al.	14 (70)	RCT	30/30 (60)	9	PSQI/ESS	Music	3 weeks	NR
Erica, et al.	15 (71)	RCT	9/9 (18)	17	EEG/HRV	Yoga Nidra	30 min	NR
Sat Bir, et al.	16 (72)	RCT	20/20 (40)	16	TEQ/ISQ/PSQI/SES/PSAS	Kundalini yoga	8 weeks	SHE
Nima, et al.	17 (73)	RCT	30/30 (60)	9	GDS/ERQ/PSQI	MBSR	8 weeks	NR

SSA, Sleep Scale A; ATFC2, activity tracker FitBit Charge 2; PSQI, Pittsburgh Sleep Quality Index; VR, Virtual Reality; DRSI, daily routine sleep intervention; HADS, Hospital Anxiety and Depression Scale; NR, Normal Routine; TWT, total wake time; PSAS, pre-sleep arousal scale; ISI, Insomnia Severity Index; MBSR, mindfulness-based stress reduction; MBTI, mindfulness-based therapy for insomnia; SM, self-monitoring; Maps, mindful awareness practices; SHE, sleep hygiene education; ESS, Epworth Sleepiness Scale; L-IE, Low-Impact Exercise; HE, Health Education; PSS, Perceived Stress Scale; CES-D, Center for Epidemiologic Studies Depression Scale; STAI, Spielberger’s State-Trait Anxiety Inventory; EEG, electroencephalography; M/MV, music/music video; PSG, polysomnography; BDI, Beck Depression Inventory; HRV, Heart rate variability; TEQ, Therapy Evaluation Questionnaire; ISQ, Insomnia Symptom Questionnaire; SES, Self-Efficacy for Sleep scale; PSAS, Pre-Sleep Arousal Scale; GDS, Geriatric Depression Scale; ERQ, the Gratz and Roemer Emotion Regulation Questionnaire; MBSR, mindfulness-based stress reduction.

*ID-60 (3) group has split into 3 different intervention therapy which is N = 19/19/16, the whole count number is 54; ID-65 (9) group has split into 3 different intervention therapy which is N = 23/24/24, the whole count number is 71; ID-69 (12) group has split into 3 different intervention therapy which is N = 35/30/29, the whole count number is 94.

Regarding the evaluation of study outcomes, the research included varied in their focus on therapeutic outcomes, hence employing diverse testing methodologies. The studies measured characteristics related to sleep quality (57, 59–73), or outcomes in other categories such as anxiety (58, 65), depression (58, 64, 68), stress (64), heart rate (71), electroencephalography (65, 71), or mood (73). The Pittsburgh Sleep Quality Index and scales were the most frequently utilized measures for assessing sleep quality. Additionally, the activity tracker FitBit Charge 2 (57), electroencephalography (65, 71), and heart rate variability (71) represented the latest measurement techniques.

An analysis using the JBI Critical Appraisal Checklist indicates that the quality of evidence from the included studies is relatively high. However, nearly all studies did not rigorously implement a double-blind experimental methodology (**Table 4**).

**Table 4:** Quality of evidence in the included 17 reports based on JBI’s critical appraisal tools.

Study Design	ID	Authors	Year	Journal	Evaluation for Evidence Reported													
					1	2	3	4	5	6	7	8	9	10	11	12	13	Ranking
RCT	1 (57)	Soon Young, et al.	2020	Intensive and Critical Care Nursing	Y	Y	Y	N	UN	N	Y	N	Y	Y	Y	Y	Y	9
RCT	2 (58)	Jennifer, et al.	2021	General Hospital Psychiatry	Y	UN	Y	N	UN	UN	N	Y	Y	Y	Y	Y	Y	8
RCT	3 (59)	Jason, et al.	2014	Sleep	Y	Y	Y	N	UN	UN	N	Y	Y	Y	Y	Y	Y	9
RCT	4 (60)	David, et al.	2015	JAMA internal medicine	Y	Y	Y	N	UN	UN	N	Y	Y	Y	Y	Y	Y	9
RCT	5 (61)	Shu-chuan, et al.	2012	The Journal of Alternative and Complementary Medicine	N	UN	Y	N	UN	UN	N	Y	Y	Y	Y	Y	N	6
RCT	6 (62)	Fuzhong, et al.	2004	Journal of the American Geriatrics Society	Y	Y	Y	N	UN	Y	N	Y	Y	Y	Y	Y	Y	10
RCT	7 (63)	Michael, et al.	2008	Sleep	Y	Y	Y	Y	UN	UN	N	Y	Y	Y	Y	Y	Y	10
RCT	8 (64)	Marı´a, et al.	2017	The Journal of Alternative and Complementary Medicine	Y	UN	Y	N	UN	UN	N	Y	Y	Y	Y	Y	Y	8
RCT	9 (65)	Chiung-Yu, et al.	2017	Complementary therapies in medicine	Y	Y	Y	N	UN	Y	N	Y	Y	Y	Y	Y	Y	10
RCT	10 (66)	Qun, et al.	2016	The Journal of Alternative and Complementary Medicine	Y	Y	Y	N	UN	Y	N	Y	Y	Y	Y	Y	Y	10
RCT	11 (67)	En-Ting, et al.	2012	International journal of nursing studies	Y	Y	Y	UN	UN	Y	Y	Y	Y	Y	Y	Y	Y	11
RCT	12 (68)	La´szlo´, et al.	2008	Journal of advanced nursing	Y	Y	Y	UN	UN	Y	Y	Y	Y	Y	Y	Y	Y	11
RCT	13 (69)	Angela, et al.	2014	Complementary therapies in medicine	Y	N	Y	N	UN	UN	Y	Y	Y	Y	Y	Y	Y	9
RCT	14 (70)	Hui-Ling, et al.	2006	Journal of advanced nursing	Y	Y	Y	N	UN	UN	Y	Y	Y	Y	Y	Y	Y	10
RCT	15 (71)	Erica, et al.	2023	Journal of Psychosomatic Research	Y	Y	Y	UN	UN	Y	Y	Y	Y	Y	Y	Y	Y	11
RCT	16 (72)	Sat Bir, et al.	2021	Journal of Clinical Sleep Medicine	Y	Y	Y	Y	Y	UN	Y	Y	Y	Y	Y	Y	Y	12
RCT	17 (73)	Nima, et al.	2024	BMC public health	Y	UN	Y	UN	UN	UN	N	Y	Y	Y	Y	Y	Y	8
Y%					94%	70%	100%	11%	5%	35%	41%	94%	100%	100%	100%	100%	94%	

JBI, the Joanna Briggs Institute; Y, Yes; N, No; UN, Unclear.

JBI Critical Appraisal Checklist for Randomized Controlled Trial: 1. Was true randomization used for assignment of participants to treatment groups? 2. Was allocation to treatment groups concealed? 3. Were treatment groups similar at the baseline? 4. Were participants blind to treatment assignment? 5. Were those delivering treatment blind to treatment assignment? 6. Were outcomes assessors blind to treatment assignment? 7. Were treatment groups treated identically other than the intervention of interest? 8. Was follow up complete and if not, were differences between groups in terms of their follow up adequately described and analyzed? 9. Were participants analyzed in the groups to which they were randomized? 10. Were outcomes measured in the same way for treatment groups? 11. Were outcomes measured in a reliable way? 12. Was appropriate statistical analysis used? 13. Was the trial design appropriate, and any deviations from the standard RCT design (individual randomization, parallel groups) accounted for in the conduct and analysis of the trial?

### 3.2 Description of the population

The comprehensive review of literature on the application of arts therapies in addressing Sleep Initiation and Maintenance Disorders (SIMDs) culminated in the selection of 17 pivotal papers. These studies are meticulously tabulated in **Tables 3**, **5**, adhering to the PICOs methodological framework, thereby illustrating the core experimental data extracted from each randomized controlled trial (RCT) article. Collectively, these papers encapsulate a population of 1,300 patients, underscoring the substantial empirical effort directed toward exploring the efficacy of diverse art therapy interventions in mitigating SIMDs.

**Table 5:** Clinical setting, country, and characteristics of the participants.

Name	Setting hospital	Nation/Region	Treatment location	Types of SIMDs	Sample size	Gender	Mean age
Soon Young, et al.	Dong-A University Hospital	South Korea	Cardiac ICU	Sleep fragmentation	48	M,F	66.42
Jennifer, et al.	Arizona State University	USA	Mobile app	Insomnia symptoms	237	M,F	44.24/44.15
Jason, et al.	Academic medical center	USA	Rush University Medical Center	Chronic Insomnia	54	M,F	42.9
David, et al.	University of California, Los Angeles	USA	UCLA medical research center	Difficulty maintaining sleep	49	M,F	66.3
Shu-chuan, et al.	two communities	Taiwan	No description	Sleep Disorders	70	F	48.60/48.69
Fuzhong, et al.	General community	USA	residential areas	No description	118	F	75.30/75.45
Michael, et al.	General community	USA	No description	Chronic Insomnia	112	F	69.6/69.7/69.8/70.7
Marı´a, et al.	the Faculty of Health Sciences of the University of Almerı´a	Spain	the Faculty of Health Sciences of the University of Almerı´a	No description	95	M,F	22.33/21.77/22.45
Chiung-Yu, et al.	No description	Taiwan	Participants’ homes	No description	71	F	41.06
Qun, et al.	Urban community centers	China	Participants’ homes	Difficulty maintaining sleep	64	M,F	69.38
En-Ting, et al.	Sleep Laboratory	Taiwan	Sleep Laboratory	Chronic Insomnia	50	M,F	31.82
La´szlo´, et al.	Semmelweis University	Hungary	Institute of Behavioural Sciences	poor sleep quality	94	M,F	22.6
Angela, et al.	community center	Singapore	Participants’ homes	poor sleep quality	60	M,F	64.0
Hui-Ling, et al.	General community	Taiwan	Participants’ homes	Trouble falling asleep	60	M,F	67
Erica, et al.	National University of Natural Medicine	USA	Helfgott Research Institute	insomnia	18	M,F	30/33
Sat Bir, et al.	Brigham and Women’s Hospital	USA	No description	sleep onset insomnia	40	M,F	43.5/40.8
Nima, et al.	No description	Iran	nursing homes	insomnia	60	M,F	66/67

Among this aggregation of research, the RCTs conducted by Jason, Chiung-Yu, and László are particularly notable for their methodological rigor. By segregating patients into three distinct groups, these studies not only facilitated a nuanced examination of the therapeutic outcomes attributable to various arts interventions and settings but also implemented strategic measures to curtail potential data bias (references 59, 65, 68). Contrasting with these, the remainder of the corpus, comprising 14 papers, predominantly adopted a dual-group treatment versus control design, which is a conventional approach within clinical trial methodologies.

Within this diverse array of studies, Jennifer’s research stands out due to its scale, encompassing a total of 237 patients divided into two groups (124:113), marking it as the study with the highest number of trials among the 17 papers reviewed (reference 58). Following closely, the works of Fuzhong and Michael also contribute significantly to the dataset with totals of 118 and 112 patients, respectively (references 62, 63). Conversely, the study led by Erica, involving a comparatively modest cohort of 18 patients, commands attention not for its volume but rather for its profound implications. Despite its smaller scale, the depth of insights and the meticulous attention to minimizing data bias within Erica’s study necessitate a detailed examination of its experimental outcomes and methodological strengths (reference 71).

### 3.3 Analysis the function with arts therapies

The main effects of each art therapy on sleep quality are shown in **Table 6**. In modern society, sleep issues have become a common health concern, not only affecting the quality of individual lives but also leading to a range of physical and mental health problems (74). As research into non-pharmacological treatments for SIMDs deepens, art therapy has gained attention as an alternative method. This review aims to explore the effectiveness and mechanisms of various arts therapies in improving sleep quality through a systematic analysis of 17 research articles.

**Table 6:** Comparative analysis of results across modalities.

Method	Name	Types	Results
meditation	Jennifer, et al. (58)	RCT	using the Calm app can lead to improvements in depression and anxiety in adults with sleep disturbance, as well as reductions in fatigue, daytime sleepiness, and pre-sleep arousal.
	Jason, et al. (59)	RCT	mindfulness meditation, especially MBSR and MBTI, significantly reduces wakefulness, pre-sleep arousal, and insomnia severity. MBTI was more effective than MBSR after 6 months, with both maintaining long-term benefits, suggesting mindfulness as a potential chronic insomnia treatment.
	David, et al. (60)	RCT	mindfulness meditation is a feasible and effective treatment option for older adults with mild sleep problems, improving sleep quality and reducing daytime impairment. The intervention had a large effect size on sleep quality.
Qigong	Shu-chuan, et al. (61)	RCT	A 12-week Qigong exercise program improved menopausal symptoms and sleep quality in perimenopausal women.
Tai Chi	Fuzhong, et al. (62)	RCT	A 6-month tai chi program significantly improves self-rated sleep quality and reduces daytime sleepiness in older adults with moderate sleep complaints.
	Michael, et al. (63)	RCT	TCC showed greater benefits compared to health education, with a higher proportion of participants achieving improved sleep quality. TCC can be considered as an effective option for enhancing sleep quality.
Biodanza	Marı´a, et al. (64)	RCT	Biodanza effectively reduces stress and depression in university students, as well as improves sleep quality. Biodanza group showed significant improvements in depression, perceived stress, and subjective sleep quality compared to the control group.
Music	Chiung-Yu, et al. (65)	RCT	music and music video interventions did not have a significant effect on objective sleep parameters, but the music group had a longer subjective total sleep time compared to the music video group.
	Qun, et al. (66)	RCT	The sustained improvement in sleep quality in the intervention group compared to the control group suggests that music intervention is an effective non-pharmacological therapy for improving sleep quality in older adults.
	En-Ting, et al. (67)	RCT	listening to soothing music improves both objective and subjective sleep quality in adults with chronic insomnia.
	La´szlo´, et al. (68)	RCT	listening to music significantly improved sleep quality in students with poor sleep, while the audiobook and control groups did not show any improvement.
	Angela, et al. (69)	RCT	After 6 weeks of listening to calming music, the intervention group had significantly better sleep quality scores than the control group.
	Hui-Ling, et al. (70)	RCT	music therapy improves sleep quality in older adults, including better perceived sleep quality, reduced sleep latency, increased sleep efficiency, less daytime dysfunction, and improved sleep duration after two weeks.
yoga	Erica, et al. (71)	RCT	Yoga Nidra is a feasible and well-tolerated intervention for individuals with insomnia, showing a potential decrease in respiratory rate.
	Sat Bir, et al. (72)	RCT	Kundalini yoga, as a primary treatment for insomnia, led to improvements in sleep onset latency and other sleep measures compared to sleep hygiene intervention, sustained at 6-month follow-up.
MBSR/MBTI	Nima, et al. (73)	RCT	The mindfulness-based stress reduction (MBSR) program led to a significant reduction in depression symptoms and improvement in emotion regulation and sleep quality among depressed elderly individuals.
VR	Soon Young, et al. (57)	RCT	VR meditation improved ICU patients’ sleep quality, including subjective sleep ratings and objective sleep measures such as shorter awake times and longer deep sleep times compared to the control group.

TCC, Tai Chi Chih; MBSR, mindfulness-based stress reduction; MBTI, mindfulness-based therapy for insomnia.

With technological advancements, meditation practices using virtual reality (VR) technology have become feasible (75). By simulating natural environments and combining deep breathing and guided meditation, VR meditation has shown significant effects in shortening the time to fall asleep, reducing the number of awakenings during the night, and increasing deep sleep duration, offering a new perspective and method for sleep therapy (57, 76).

Meditation, especially mindfulness meditation, has proven to be an effective self-regulation method for chronic insomnia sufferers by reducing anxiety and stress before sleep, shortening the time to fall asleep, and improving sleep quality (77). Research indicates that meditation not only alleviates SIMDs but also reduces negative emotions such as anxiety and depression, thereby enhancing daytime function and quality of life. These multiple benefits make meditation a powerful tool for treating SIMDs (59).

Qigong and Tai Chi, ancient Chinese mind-body practices (78), improve sleep quality through a series of slow, orderly movements and breathing techniques. These practices not only promote physical relaxation and reduce psychological stress but also regulate the endocrine system, effectively improving SIMDs (79). For example, a 12-week Qigong training for premenopausal women successfully improved their sleep quality and daytime drowsiness (61). Similarly, Tai Chi practice has been shown to enhance self-reported sleep quality and physical function in the elderly, indicating its positive effect on sleep improvement (62).

Biodanza therapy, a dance movement therapy that integrates music, movement, and emotional expression (64), did not show significant differences in directly improving sleep quality but effectively alleviated stress and depression, indirectly promoting a better rest state. Its significant effect in relieving stress, anxiety, and depression indirectly helps attain better sleep (80). Music therapy, including listening to soothing music and music videos, has also shown potential in improving sleep quality in adults and the elderly (65). Music can extend subjective sleep duration, shorten stage two sleep duration, prolong rapid eye movement sleep, and overall improve participants’ sleep experience (67, 81). These studies emphasize music as a non-pharmacological intervention that improves sleep quality by reducing stress responses, relaxing the body and mind, and diverting attention (82).

Yoga therapy, particularly Kundalini yoga and Yoga Nidra, has been proven to have a positive impact on improving sleep quality (71, 72). By improving physical flexibility, enhancing muscle strength, deep breathing, and meditation practices, it not only improves sleep quality but also has a significant therapeutic effect on emotional disorders such as depression and anxiety (83). These improvements suggest that yoga can be an effective treatment method for SIMDs. This effectiveness is not only immediate at the end of yoga practice but can last up to six months after the practice has ended (84), implying that the improvement in sleep quality through yoga is not temporary but has a certain durability. This underscores yoga practice as a long-term effective non-pharmacological treatment method, not just a short-term intervention.

Mindfulness-Based Stress Reduction (MBSR)/Mindfulness-Based Cognitive Therapy (MBCT) courses have had a positive effect on improving emotional regulation and sleep problems (73), significantly reducing depressive symptoms and improving sleep quality in the elderly population with depression.

Overall, these arts therapies provide a diverse range of non-pharmacological treatment options for SIMDs. They significantly improve sleep issues through various mechanisms such as emotional regulation, stress reduction, improving the wakefulness state before sleep, and enhancing sleep quality. Future research should further explore the specific mechanisms of action, sustained effects, and how these arts therapies can be effectively integrated into existing sleep disorder treatment frameworks.

### 3.4 Literature quality assessment

Leveraging **Table 4**, our analysis meticulously appraised the comprehensive quality of each study through the Joanna Briggs Institute (JBI) methodology, assigning a one-point increment for every “Yes” response, culminating in an aggregate score out of 13. Within this cohort of 17 randomized controlled trials (RCTs), the paper authored by Sat Bir distinguished itself with a high-quality score of 12 (reference 72), closely trailed by the contributions from En-Ting, László, and Erica, each securing a commendable score of 11 (references 67, 68, 72). This echelon of papers stands apart for their rigorous alignment with quality parameters, whereas the corpus of remaining studies predominantly manifested moderate quality, with the exception of Shu-chuan’s work, which uniquely aligns with the aforementioned group by also securing a score of 11 (references 67, 68, 72). One RCT article notably diverged from this trend, recording a modest score of 6 (reference 61) upon JBI evaluation.

A deeper dive into the JBI quality assessment criteria across these 17 RCT articles revealed that questions 3, 9, 10, 11, and 12 witnessed unanimous affirmative responses. This uniformity underscores a foundational concordance among the studies with regards to the critical aspects of including treatment groups based on clear baseline characteristics, ensuring randomness in subgroup allocations, maintaining uniformity and authenticity in measurement techniques, and employing appropriate statistical analyses. Such consistency is pivotal for establishing the foundational integrity of trial methodologies.

Conversely, a nuanced examination reveals that questions 4 and 5, which probe the implementation of double-blind or single-blind protocols in RCTs, presented a spectrum of “No” and “Unclear” responses across all examined literature. This variance highlights a prevalent methodological vulnerability within the domain, signifying a crucial area for future methodological refinement and adherence to ensure the elimination of bias and elevation of research quality standards.

### 3.5 Description of the control intervention

According to **Table 3**, within the ambit of the 17 randomized controlled trials (RCTs) scrutinized in the presented literature, a discernible allocation of control group methodologies is observed. Notably, a majority, specifically 10 RCTs, embraced a ‘Normal Routine’ strategy for their control groups, an approach characterized by the absence of any specialized methodological intervention for participants afflicted with sleep disorders (58, 61, 64, 65, 67–71, 73). This strategy ostensibly serves to mirror the unaltered daily routines of individuals, thereby establishing a baseline for comparative analysis. An integrative approach was adopted in three RCTs, employing ‘Sleep Hygiene Education’ as the control group intervention. This methodological choice is predicated on the dissemination of essential medical knowledge, aiming to cultivate an awareness among patients regarding the principles conducive to healthy sleep patterns (60, 66, 72).

Complementing these, the remainder of the dataset, comprising four RCTs, instituted a varied spectrum of control conditions, specifically ‘Daily Routine Sleep Intervention,’ ‘Self-Monitoring,’ ‘Low-Impact Exercise,’ and ‘Health Education.’ These interventions (57, 59, 62, 63) were distinctively implemented.

### 3.6 Main results on the primary outcomes

Under PICO(S) design in **Table 3**, In the analytical examination of 17 randomized controlled trials (RCTs) delineated in our systematic review, the utilization of the Pittsburgh Sleep Quality Index (PSQI) emerged as the predominant tool for outcome measurement, with an overwhelming 14 studies (57, 60–70, 72, 73) electing the PSQI as their principal evaluative instrument. This universal adoption underscores the PSQI’s acknowledged efficacy in gauging the multifaceted aspects of sleep quality within clinical research paradigms. The Pre-Sleep Arousal Scale (PSAS) was employed as the measurement methodology in two distinct RCTs (59, 72), signifying its specialized application in assessing pre-sleep cognitive and somatic arousal levels. Of note, one study (57) innovatively combined the PSQI with both the Sleep Scale A (SSA) and an activity tracker (FitBit Charge 2, ATFC2), thus broadening the spectrum of sleep-related data acquisition. Similarly, an additional study (59) integrated Total Wake Time (TWT) alongside the PSQI, thereby enriching the dimensional coverage of sleep disturbances being investigated.

The Epworth Sleepiness Scale (ESS), a tool designed to measure daytime sleepiness, was cohesively utilized across three studies (62, 68, 70). Notably, the sophisticated technology of electroencephalography (EEG) was harnessed for data collection in two studies (65, 71).

### 3.7 Therapeutic with SIMDs

Music therapy, featured prominently as the most frequently cited intervention method among the 17 studies reviewed, distinguishes itself from other modalities by its passive nature, as opposed to the active engagement required by the others (65–70). This form of therapy, characterized by the passive auditory reception of music, serves to alleviate emotional tension and soothe neural stress without necessitating physical movement, making it particularly well-suited for improving sleep disturbances among the elderly with limited mobility. According to **Table 4** of the PICO(S) framework, in the elderly population, two randomized controlled trials (RCTs), each involving around 60 participants split into two groups, conducted interventions over periods of 3 and 6 weeks. The pre and post-intervention data, gauged by the Pittsburgh Sleep Quality Index (PSQI), demonstrated a comprehensive amelioration of SIMDs in the elderly (69, 70). In studies targeting younger demographics, such as college students, La´szlo´ divided participants into three groups of 35, 30, and 29. After a 3-week intervention, data from the PSQI and Beck Depression Inventory (BDI) indicated that music therapy could concurrently suppress depressive symptoms and sleep disorder manifestations (68). A 3-day experiment focusing on adults with chronic insomnia revealed that listening to soothing music for 45 minutes before sleep significantly prolonged the rapid eye movement (REM) sleep phase (67). As a form of passive intervention, music therapy, a non-pharmacological measure, has been shown to effectively enhance sleep initiation and maintenance in patients with sleep quality issues (85), particularly benefiting those who prefer natural remedies or are concerned about the side effects of sleep medications, without requiring physical movement (86). Allowing participants to select their preferred music enhances the effectiveness of the intervention, as personal preference plays a critical role in the therapeutic impact of music. However, choices in music can vary greatly, and there is no standardized method for selecting the type or duration of music to be used for sleep therapy. Nevertheless, according to **Table 4** of the PICO(S) framework, most studies on music therapy interventions had short durations, lacking data on the long-term efficacy and sustainability of music therapy for treating SIMDs (65, 67, 68, 70).

Besides music therapy’s passive interventions, all other methods included in the 17 studies fall under active interventions, with meditation and mindfulness being primary modalities (57–60, 73), followed by physical movement-based interventions like Qigong, Tai Chi, dance, and yoga (61–64, 71, 72). Tai Chi and Qigong therapies, among these active interventions, had the longest durations of engagement, spanning 2 months, 3 months, and 6 months, utilizing slow, rhythmic movements and deep breathing to achieve relaxation and equilibrium of body and mind. Studies have shown that Qigong improved menopausal symptoms and sleep quality in the intervention group after 6 and 12 weeks compared to the control group, while Tai Chi required long-term intervention, such as more than 24 weeks, to exhibit a significant improvement in sleep, with the Pittsburgh Sleep Quality Index (PSQI) and Epworth Sleepiness Scale (ESS) serving as primary measurement tools (61–63). Meditation is a commonly employed method in the treatment of SIMDs, typically involving 6–8 week interventions focused primarily on elderly individuals, especially those experiencing sleep disturbances. Research suggests that mindfulness meditation may correlate with reduced concentrations of NF-κB (a transcription factor associated with inflammatory responses), indicating a potential anti-inflammatory effect beneficial to seniors. Furthermore, meditation therapy has been found to improve mental health issues in the elderly, alleviating depression, anxiety, and feelings of loneliness. Meditation, often linked to Buddhism or yoga, typically incorporates techniques such as silence, breath counting, and visualization during training, aimed at reducing stress and intrusive thoughts, thereby psychologically remedying non-organic insomnia (58–60, 73). Yoga and dance, requiring some degree of physical effort, have shown varying impacts. Despite no significant intergroup differences in EEG alpha-wave power, heart rate variability (HRV), or sleep latency observed with Yoga Nidra (a form of guided meditation practice), a statistically significant difference in respiratory rate between the Yoga Nidra group and the control group suggests that Yoga Nidra may aid in relaxation, though further research is necessary to confirm (71). The effects of Kundalini yoga and Sleep Hygiene Education (SH) on chronic primary insomnia showed the yoga group had greater improvements in sleep onset latency (SOL), total sleep time (TST), and sleep efficiency (SE), with these improvements persisting at a 6-month follow-up. Over 50% of participants in the yoga group reported a decrease of at least 8 points in the Insomnia Severity Index (ISI) at the end of treatment and during the follow-up period (72). The dance therapy Biodanza, after a 4-week intervention among university students, significantly reduced stress, tension, and depressive moods, indirectly ameliorating sleep issues to some extent (64).

Virtual reality (VR) devices, with their immersive capabilities, offer a promising complement to meditation or mindfulness therapies. However, caution is warranted when applying this emerging technology to older populations, as it may induce fear, stress, and anxiety, potentially exacerbating sleep disorder issues (57).

In conclusion, music therapy emerges as the most extensively documented passive intervention within the study, utilizing auditory reception to mitigate emotional and stress-related issues. It has proven effective in improving sleep disturbances among both the elderly and younger populations, requiring no physical exertion and allowing for personalized music selection to enhance intervention outcomes. Despite the absence of data on long-term effectiveness, music therapy remains a beneficial non-pharmacological intervention (87). Concurrently, the study also explores active interventions such as meditation, Qigong, Tai Chi, yoga, and dance, which are also effective in improving sleep quality among specific groups. The nascent combination of VR technology with meditation shows potential therapeutic effects but should be approached cautiously when applied to older individuals to avoid eliciting adverse emotional responses.

## 4 Discussion

Given the comprehensive analysis of the results surrounding different arts therapies for mitigating SIMDs, our discussion will explore the optimal combination of these therapies to achieve maximum effectiveness for various demographics. The results highlight the varying benefits of passive and active interventions, including music therapy, meditation, Tai Chi, Qigong, Yoga, and Biodanza, as well as emerging technologies like virtual reality (VR) meditation. By understanding the unique advantages of each method, we can propose tailored, multimodal therapy regimens that cater to specific needs.

The evidence strongly suggests that a combination of passive and active therapies might offer a synergistic effect on improving sleep quality and duration (88–90). Music therapy, the most extensively studied passive intervention in the included literature, demonstrated significant potential in improving sleep quality for both elderly and younger populations without the necessity of physical movement (91, 92). This points to the utility of integrating music therapy into the bedtime routine of individuals across different age groups, particularly those with limited physical mobility or preference for less physically demanding interventions (93, 94).

On the other hand, active therapies like Meditation, Tai Chi, Qigong, and Yoga, have shown promise in not only improving sleep quality but also enhancing psychological well-being and reducing stress, anxiety, and depressive symptoms. These findings suggest that active interventions, which often incorporate elements of mindfulness, physical movement, and breathwork, can address both the physiological and psychological contributors to SIMDs.

The diversity in demographics across the studies, including varying age groups, health statuses, and cultural backgrounds, underscores the necessity of personalized therapy plans. For instance, older adults may benefit more from a combination of music therapy with gentle Qigong exercises, which mitigate the risk of falls while promoting relaxation and improving sleep (95). Conversely, younger individuals, particularly those experiencing stress and anxiety (e.g., college students), might find a combination of high-intensity Biodanza and meditation more effective, leveraging the physical exertion of dance to alleviate stress and the mindfulness aspect of meditation to prepare the mind for rest (96, 97).

Additionally, for those experiencing more profound psychological issues, such as depression, integrating therapies that specifically target emotional well-being—like meditation with music therapy (98)—can be particularly beneficial. This combination can leverage the stress-reducing and mood-enhancing benefits of music alongside the mindfulness and self-awareness cultivated through meditation, offering a holistic approach to treating SIMDs.

The integration of VR technology into meditation practices presents an innovative approach to enhancing the immersion and effectiveness of mindfulness exercises for SIMDs. However, given the potential for VR to induce anxiety or discomfort in certain populations, its application should be carefully considered. Tailoring VR experiences to match individual preferences and introducing these technologies gradually can mitigate potential adverse reactions. Furthermore, combining VR-enhanced meditation with more traditional, familiar therapeutic practices like music therapy could offer a balanced approach, providing the novel benefits of VR while ensuring comfort and accessibility (99).

For the integration of arts therapies to be most effective, ongoing assessment and adaptation of therapy plans are crucial. This includes regular monitoring of individual responses to therapy, readiness to modify or switch therapies based on efficacy and preference, and continually updating the therapy plan as new evidence emerges. Future research should focus on longitudinal studies to better understand the long-term effectiveness of combined therapies and to refine these recommendations further.

SIMDs frequently coexist with, or are exacerbated by, psychological conditions such as anxiety, depression, and stress, suggesting a bidirectional relationship between sleep disturbances and mental health issues (100). The complex interplay between these factors highlights the importance of emotional regulation in the effective management of SIMDs. As sleep problems often serve as both a symptom and a contributor to mental health issues, addressing the psychological underpinnings is paramount (101). This underscores the necessity for therapeutic approaches that not only target the physiological aspects of SIMDs but also focus on psychological de-escalation. Arts therapies, with their inherent capacity for emotional expression and regulation, offer a unique conduit for this purpose (102). In the forward trajectory of research, it is imperative that experimental designs embody stringent rigor, with the incorporation of single-blind and double-blind methodologies in randomized controlled trials (RCTs) being non-negotiable to mitigate the potential for data skewness. Furthermore, the endeavor to extend art therapy over protracted durations for the amelioration of sleep disorders poses a formidable challenge in the context of healthcare personnel ratios. The paucity of healthcare professionals in numerous developing nations presents a tangible barrier to the scalable application of art therapy to a broad demographic afflicted with sleep disturbances. This scenario necessitates a considerable investment of resources and dedication, thereby underscoring the exploration of artificial intelligence (AI) as a plausible adjunct or enhancer of art therapy practices. The potential for AI to bridge the gap in patient care and augment the efficacy of arts therapies represents a nascent yet promising paradigm, meriting earnest scholarly attention.

## 5 Limitations

The systematic review of randomized controlled trials (RCTs) evaluating the efficacy of arts therapies in addressing SIMDs provides strong evidence supporting the potential advantages of these interventions. Nonetheless, the presence of several methodological limitations within the analyzed studies necessitates thorough scrutiny, as it could affect the interpretation of results and guide the trajectory of subsequent research in this domain.

The systematic review highlights a prevalent issue in the included randomized controlled trials (RCTs) regarding participant blinding and the blinding of individuals administering treatments. As identified using the Joanna Briggs Institute (JBI) critical appraisal tool, a majority of the studies (11 out of 17) lacked participant blinding, raising serious concerns about bias in self-reporting of symptoms and treatment response. This lack of blinding could lead participants to have heightened expectations or placebo effects, which are well-documented phenomena in clinical research. These biases can significantly skew the reported efficacy of the arts therapies, leading to potentially inflated outcomes.

The absence of blinding among treatment administrators in 16 out of the 17 reviewed studies poses a critical threat to the validity of the study findings. Unblinded facilitators might unconsciously convey their expectations to participants, affecting the latter’s responses and engagement. This methodological oversight complicates the interpretation of the efficacy of arts therapies, as the observed benefits could be attributed to non-specific effects rather than the therapeutic intervention itself. Restricting the systematic review to English-language studies introduces another significant limitation by potentially overlooking valuable research conducted in other languages. This limitation not only restricts the diversity of the data but also introduces a cultural bias, limiting the generalizability of the findings to English-speaking populations. Art therapy practices, being deeply influenced by cultural contexts, might show different levels of efficacy and engagement in diverse cultural settings. Therefore, extending future systematic reviews to include multiple languages and cultural contexts could uncover more nuanced insights about the applicability and effectiveness of arts therapies across different global populations.

The short duration of interventions reported in four of the RCTs presents another limitation regarding the sustainability of the therapy benefits. While immediate improvements in sleep quality were noted, the long-term efficacy of these interventions remains uncertain. This limitation is significant as the chronic nature of SIMDs requires sustained management strategies. Future research should focus on longitudinal studies that assess the effects of arts therapies over extended periods to better understand and validate the longevity of the therapeutic benefits.

## 6 Conclusion

This comprehensive review, through an extensive analysis of 17 studies, examines the efficacy and mechanisms of arts therapies—including music therapy, meditation, Tai Chi, Qigong, yoga, Biodanza, and VR meditation—in improving Sleep Initiation and Maintenance Disorders (SIMDs). The findings reveal that these arts therapies, whether employed singularly or in conjunction, serve as effective non-pharmacological interventions to enhance sleep quality. Notably, music therapy, as a passive modality, significantly improves sleep quality in both elderly and younger populations, indicating its suitability as a pre-sleep routine across various age groups. Active therapies such as meditation, Tai Chi, Qigong, and yoga not only aid in augmenting sleep quality but also demonstrate positive effects on mental health, offering relief from stress, anxiety, and symptoms of depression. These active interventions, integrating mindfulness, physical movements, and breathing techniques, provide a holistic approach to addressing both physiological and psychological factors associated with SIMDs. The research underscores the importance of tailoring personalized arts therapy programs based on age, health status, and cultural backgrounds—for instance, combining music therapy and Qigong to improve sleep in the elderly, while Biodanza and meditation are more effective in stress relief among the younger demographic. Integrating new technologies like VR with traditional therapies offers innovative experiences in treating sleep disorders. Specific to practical applications in existing medical protocols, integration strategies such as professional training on arts therapies and infrastructural enhancements in healthcare settings can facilitate their adoption. Challenges such as cultural and institutional resistance, alongside hurdles in cost and resource allocation, may impede implementation, yet growing patient preference for non-drug treatments and supportive research outcomes provide favorable conditions for their integration. Future research should focus on the long-term effects of these interventions, integration of technological innovations like artificial intelligence, and the execution of cross-cultural studies to better understand global applicability and cultural influences on therapy effectiveness. Although this review confirms the effectiveness of arts therapies in treating SIMDs, future research should focus on the longevity of therapeutic outcomes, refinement of personalized treatment plans, and innovation through new technology integration, emphasizing longitudinal study designs to understand the long-term effects of combined therapies more comprehensively and further refine treatment recommendations.

## Data availability statement

The original contributions presented in the study are included in the article/supplementary material. Further inquiries can be directed to the corresponding author.

## Author contributions

XL: Conceptualization, Data curation, Formal analysis, Funding acquisition, Investigation, Methodology, Project administration, Resources, Software, Supervision, Validation, Visualization, Writing – original draft, Writing – review & editing. AZ: Writing – original draft, Writing – review & editing. HL: Writing – original draft, Writing – review & editing. YL: Conceptualization, Data curation, Formal analysis, Writing – review & editing. FY: Funding acquisition, Writing – review & editing. XW: Data curation, Software, Writing – review & editing. QY: Data curation, Software, Writing – review & editing. ZZ: Investigation, Writing – review & editing. GH: Conceptualization, Data curation, Formal analysis, Funding acquisition, Investigation, Methodology, Project administration, Resources, Software, Supervision, Validation, Visualization, Writing – original draft, Writing – review & editing.
